# Analytical Approach to Study Sensing Properties of Graphene Based Gas Sensor

**DOI:** 10.3390/s20051506

**Published:** 2020-03-09

**Authors:** Ali Hosseingholipourasl, Sharifah Hafizah Syed Ariffin, Yasser D. Al-Otaibi, Elnaz Akbari, Fatimah. KH. Hamid, S. S. R. Koloor, Michal Petrů

**Affiliations:** 1 UTM-MIMOS Center of Excellence in Telecommunication Technology, School of Electrical Engineering, Universiti Teknologi Malaysia, Skudai 81310, Johor, Malaysia; poorasl.ali@gmail.com (A.H.); shafizah@utm.my (S.H.S.A.); 2 Faculty of Computing and Information Technology in Rabigh, King Abdulaziz University, Jeddah 21589, Saudi Arabia; yalotaibi@kau.edu.sa; 3 Department for Management of Science and Technology Development, Ton Duc Thang University, Ho Chi Minh City 758307, Vietnam; 4 Faculty of Electrical and Electronics Engineering, Ton Duc Thang University, Ho Chi Minh City 758307, Vietnam; 5 School of Electrical Engineering, Universiti Teknologi Malaysia, Skudai 81310, Johor, Malaysia; fatimahkhairiah@gmail.com; 6 Institute for Nanomaterials, Advanced Technologies and Innovation, Technical University of Liberec, Studentska 2, 461 17 Liberec, Czech Republic; s.s.r.koloor@gmail.com (S.S.R.K.); michal.petru@tul.cz (M.P.)

**Keywords:** graphene, gas sensor, adsorption, *I-V* characteristics, analytical modeling

## Abstract

Over the past years, carbon-based materials and especially graphene, have always been known as one of the most famous and popular materials for sensing applications. Graphene poses outstanding electrical and physical properties that make it favorable to be used as a transducer in the gas sensors structure. Graphene experiences remarkable changes in its physical and electrical properties when exposed to various gas molecules. Therefore, in this study, a set of new analytical models are developed to investigate energy band structure, the density of states (DOS), the velocity of charged carriers and *I-V* characteristics of the graphene after molecular (CO, NO_2_, H_2_O) adsorption. The results show that gas adsorption modulates the energy band structure of the graphene that leads to the variation of the energy bandgap, thus the DOS changes. Consequently, graphene converts to semiconducting material, which affects the graphene conductivity and together with the DOS variation, modulate velocity and *I-V* characteristics of the graphene. These parameters are important factors that can be implemented as sensing parameters and can be used to analyze and develop new sensors based on graphene material.

## 1. Introduction

Electronics and sensors are required to provide valuable advantages for humanity in different vital areas such as life sciences, health care, and security. Electrical sensors are electronic devices capable of detecting physical quantities in their environment and convert them into measurable electrical signals that can be displayed by the monitor [1,2,3]. Sensors can be designed, fabricated, and employed for various applications subject to the physical quantity to be measured [4,5,6,7,8]. Depending on the type, sensors can measure specific changes in the properties of different materials such as liquids and gases. One of the most important applications of sensors is the detection of specific gases [9,10,11]. There are a variety of harmful and toxic gases threatening human life in many working and living environments [12]. Sensitive devices are required to detect and monitor the gaseous threats that are difficult to detect with our own senses or see by the naked eye. 

Over the last decade, graphene as a two-dimensional material has been suggested and studied for various practical applications. It has unique electrical, chemical, and structural properties; therefore, graphene outperforms its counterparts for sensing applications [13,14,15]. In terms of chemical properties, graphene shows very high chemical stability. The low power consumption, high sensitivity, the fast response time (sensor response in less than 10 s), as well as short recovery time were reported as advantages offered by graphene-based sensors [16]. Electrically, graphene is very sensitive to its environment and adsorption/desorption of charge [17,18]. The ideal 2D structural system of graphene allows it to have very high electron and hole mobility [19,20,21]. Furthermore, experimental studies indicated that graphene sensors could operate and detect various gas molecules such as CO, NO_2_, H_2_O, and NH_3_ at room temperature [22,23,24]. So far, numerous studies have been conducted regarding the fabrication and characterization of graphene and its physical and electronic characteristics have been studied and analyzed [25,26,27]. In addition, various experimental works practically focused on the fabrication of graphene-based biosensors and gas sensors; and the significance of graphene-based electrodes, graphene functionalization techniques, and the detection mechanisms of the various target analytes have been investigated previously [28,29,30,31,32]. There are many challenges in the synthesis and fabrication of the sensors, especially nanosensors. The experimental sensing approaches suffer some restrictions and drawbacks, such as being time-consuming, high costs, and complexity of mechanism [33,34]. The use of theoretical and analytical methods is an economical and proper way to overcome the limitations of practical methods. On the other hand, many theoretical and modeling studies have been conducted regarding the modeling of gas sensors using graphene and other nanomaterials. Some of these studies have used completely similar approaches in the modeling process as they introduced some fitting parameters to fit the suggested models to experimental data [35,36,37,38]. They did not provide physical and mathematical justification for fitting parameters used in the model. In some studies, the Tight-Binding technique was used for modeling of carbon nanomaterial-based devices and sensors, but only statistical states have been considered in the calculation, and the non-equilibrium conditions were taken into account in the mathematical formulations [39,40,41,42,43]. Therefore, the main objective of this study is to analytically model and investigate the use of graphene-based field effect transistor (GFET) as gas sensors. The analytical approach consists of (a) describing the graphene with a tight-binding Hamiltonian, (b) calculating band structure, the density of states and electron velocity, (c) using the *I-V* formula to calculate the transfer characteristics of transistors with graphene as a channel. These properties are important factors that can be used to monitor sensor properties and investigate its performance against the adsorption of gas molecules. Sensor modeling can guide the experiments by showing the possible behavior and how these behaviors depend on the methods that are chosen in simulation.

It has been proven that the electrical and physical properties of the graphene indicate outstanding variation after exposure to the various target analyte of the gas molecules. These variations can be implemented as sensing parameters to monitor adsorption of various gases on the graphene surface and develop sensors with high selectivity and sensitivity.

**Figure 1:**
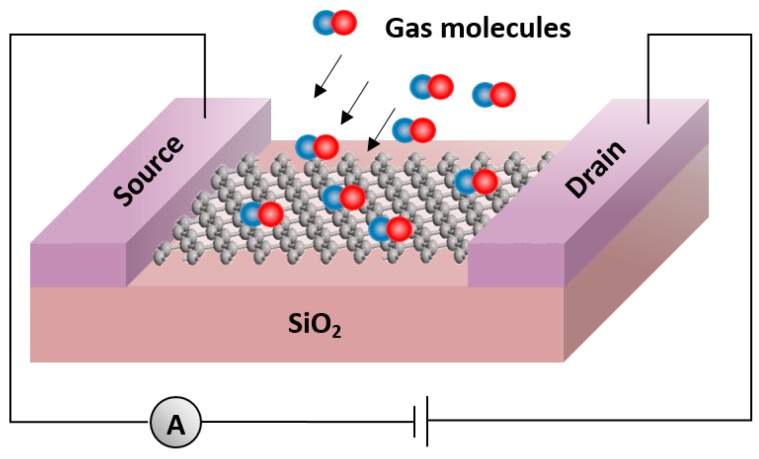
Schematics of a graphene field-effect transistor-based gas sensor.

The schematic of a graphene field-effect transistor-based sensor is illustrated in Figure 1. When gas molecules come to the graphene surface, they can affect and change both the electrical and physical properties of graphene. The atomic forces between the carbon atoms of graphene and the adsorbate can affect the graphene’s energy band structure and adjust its energy bandgap.

This leads to the transition of graphene from metallic to semiconducting energy state and modifies the electrical conductance of the graphene channel in the graphene-based FET. The changes in the energy band structure, bandgap, and the number of charged carriers in the transistor channel lead to modify the density of stats, carrier concentration, and thus, carrier velocity, resulting from modulating the current-voltage properties of the sensor.

The presented study is somehow analogous to our previous work [44] but has main differences based on sensor substrate structure and introducing the on-site energy parameter, $E_{0}^{\prime }$, for the adsorbed gas molecules. In terms of the structure, graphene is used as a substrate that provides a higher surface to volume ratio, which is more favorable for sensing applications. Graphene has a 2D structure (GNR is 1D). Thus, the number of molecules that can be adsorbed on graphene is higher, which means graphene provide a large detection range. There are some benefits to use graphene instead of the GNR regarding fabrication process in terms of cost and complexity.

On the other hand, in the previous model, the on-site energies of the adsorbed gas molecules were not considered in the modeling, and the energies of the carbon atoms of the GNR and adsorbed molecules were considered to be the same. But in the current models, we differentiate between different types of gas molecules and with carbon atoms of the graphene.

Here, three random gases CO, NO_2_, H_2_O, are chosen to be applied for sensing purposes. These three gasses are famous gases that are extensively being used in industrial and medical applications. We use three different gases to evaluate the proposed models and to show that the proposed models can predict different types of gas molecules. In addition, we used perfect graphene for mathematical formulation and modeling of the gas sensor. However, for future studies, the current modeling technique can be modified to apply for the defected graphene as well, as defected graphene can provide higher sensitivity and faster response and recovery time [45,46].

## 2. Materials and Methods

In the graphene energy band structure, the valence and conduction bands overlap in Dirac points that makes graphene to have no energy gap [47]. The electrical and physical properties of the graphene are dependent and specified mostly based on hopping energies and on-site energies of carbon atoms. On the other hand, in the graphene energy band structure, the highest valence band and the lowest conduction band are the main responsible for the conductivity and electrical characteristics [48]. Thus, in our model, only these two bands are considered in the calculations. Based on this concept, we start with the modeling of the graphene energy band structure around the Fermi energy level.

### 2.1. Energy Band Structure Modeling

**Figure 2:**
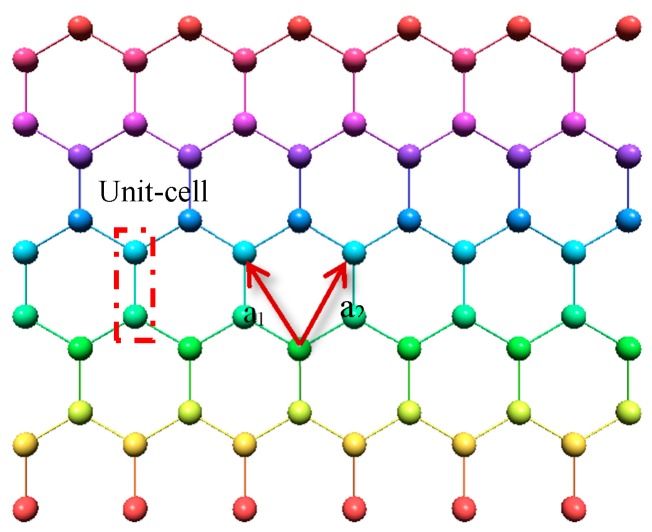
Schematics geometry of the 2D-graphene layer.

To calculate the low energy band for graphene, the tight-binding technique is adapted. In our tight-binding model, graphene as a 2D material is incorporated with the assumption that there is only one orbital per atom leading to the ultimate matrix form of the Schrödinger Equation as [48]:

(1) $$\sum_{m}\left[H_{nm}\right]\left\{\varphi_{m}\right\}=E\left\{\varphi_{n}\right\}$$

where *H_nm_* is the Hamiltonian operator matrix, *φ_n_*_or m_ is the column vector representing the wave function in the unit-cell *n* or *m*, and *E* is energy [49]. The schematics of the graphene structure with a pair of carbon atoms per unit-cell is shown in Figure 2. 

To calculate the energy dispersion relation for graphene, we need to solve the above Schrödinger Equation. Thus, first, we should acquire Hamiltonian matrixes for the graphene unit cells. Each graphene unit-cell (n^th^ unit cell) has four closest neighbor unit cells. In addition, each unit cell consists of two carbon atoms. Therefore, based on the theory that employs a single orbital per carbon atom, a (2 × 2) Hamiltonian matrix indicating the valence and conduction band in the energy band structure can be achieved. In the Hamiltonian matrixes, the upper or lower diagonal matrix elements for the neighbor carbon atoms of the graphene are assumed as *t*, while the rest of them are zeros.

(2) $$h(k)=\left(\begin{array}{cc} E_{0} & t\\ t & E_{0} \end{array}\right)+\left(\begin{array}{cc} 0 & t\\ 0 & 0 \end{array}\right)e^{-i\vec{k}.{\vec{a}}_{2}}+\left(\begin{array}{cc} 0 & 0\\ t & 0 \end{array}\right)e^{i\vec{k}.{\vec{a}}_{1}}+\left(\begin{array}{cc} 0 & 0\\ t & 0 \end{array}\right)e^{i\vec{k}.{\vec{a}}_{2}}+\left(\begin{array}{cc} 0 & t\\ 0 & 0 \end{array}\right)e^{-i\vec{k}.{\vec{a}}_{1}}$$

Defining:

(3) $$h_{0}=t+te^{i\vec{k}.{\vec{a}}_{2}}+te^{i\vec{k}.{\vec{a}}_{1}}=t+2te^{ik_{x}a}\cos(k_{y}b)$$

where $a=3a_{0}/2$ and $b=\sqrt{3}a_{0}/2$, *a_1_* and *a_2_* are the lattice vectors, *k_x_* and *k_y_* are the unit vector components, *t* represents the hopping energy parameter of graphene atoms, and *E_0_* is the on-site energy of a carbon atom. Finally, the Eigen equation for the Eigen value *E* can be obtained as [48]:

(4) $$E=E_{0}+\left|h_{0}\right|=E_{0}\pm t\sqrt{1+4\cos(k_{x}a)\cos(k_{y}b)+4\cos^{2}(k_{y}b)}$$

**Figure 3:**
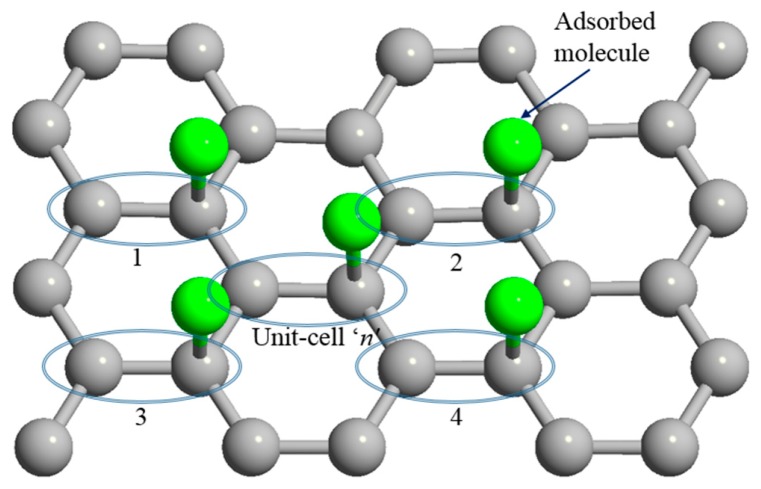
Molecular adsorption on graphene’s n^th^ unit cell and neighbor unit cells.

Once the impurity molecules adsorbed on graphene, the on-site energies of the adsorbed molecules and the hopping energies between gas molecules and graphene, contribute to the graphene characteristics and modify its electrical and physical properties such energy band structure, density of states, concentration, and velocity of carriers and current-voltage properties. To model gas effects on these parameters, the tight-binding (TB) approach based on the nearest neighborhood is employed for the graphene in the presence of the gas molecule. This time, our Hamiltonians will be (3 × 3) matrixes in the presence of the gas molecules. On the other hand, in our Hamiltonians, a single contact between the adsorbate and carbon atom is presumed as depicted in Figure 3.

Similar to Equation (2), according to the tight-binding model based on the nearest neighbor approximation, the Hamiltonian matrixes for the unit cell ‘*n*’ and its four nearest neighbors considering the adsorbed molecule can be described as:

(5) $$h(\vec{k})=\left(\begin{array}{ccc} E_{0} & t & 0\\ t & E_{0} & t^{\prime }\\ 0 & t^{\prime } & E_{0}^{\prime } \end{array}\right)\times e^{ik\left(d-d\right)}+\left(\begin{array}{ccc} 0 & 0 & 0\\ t & 0 & 0\\ 0 & 0 & 0 \end{array}\right)\times e^{i\vec{k}a_{1}}+\left(\begin{array}{ccc} 0 & 0 & 0\\ t & 0 & 0\\ 0 & 0 & 0 \end{array}\right)\times e^{i\vec{k}a_{2}}+\left(\begin{array}{ccc} 0 & t & 0\\ 0 & 0 & 0\\ 0 & 0 & 0 \end{array}\right)\times e^{-i\vec{k}a_{1}}+\left(\begin{array}{ccc} 0 & t & 0\\ 0 & 0 & 0\\ 0 & 0 & 0 \end{array}\right)\times e^{-i\vec{k}a_{2}}$$

where $E_{0}$ and $E_{0}^{\prime }$ are the on-site energies of the carbon atom and the adsorbed molecule, and $t^{\prime }$ the hopping energy between the adsorbate and a carbon atom.

By summation of individual matrixes over the *n^th^* unit cell and its four neighbors, and then calculating the determinants of the *h(k)*, the energy dispersion model for graphene, considering the molecular adsorption effects, is achieved as:

(6) $$\left|h(\vec{k})\right|=\left|\left(\begin{array}{ccc} E_{0} & t+te^{-ika_{1}}+te^{-ika_{2}} & 0\\ t+te^{ika_{1}}+te^{ika_{2}} & E_{0} & t^{\prime }\\ 0 & t^{\prime } & E_{0}^{\prime } \end{array}\right)\right|$$

(7) $$E=\frac{1}{2}\left(\pm \sqrt{{\left(E_{0}+E_{0}^{\prime }\right)}^{2}-4E_{0}E_{0}^{\prime }+4t^{\prime^{2}}+4t^{2}+16t^{2}\cos^{2}\left(k_{y}b\right)\cos\left(k_{x}a\right)}+(E_{0}+E_{0}^{\prime })\right)$$

where the on-site energy of the adsorbed molecule is represented by $E_{0}^{\prime }$ and *t’* is the hopping integral parameter that shows the hopping energy between the carbon atom and the adsorbate. The value of the *E_0_* is set to be zero as the origin of the energy [42]. The value of the t is fixed to be t = 2.7 eV, but *t’* will have different values depending on the nature of the target gas molecule. In the case of the graphene without gas, the value of the *t’* is zero; by tuning it to non-zero quantities, the gas adsorption effects can be applied. 

**Figure 4:**
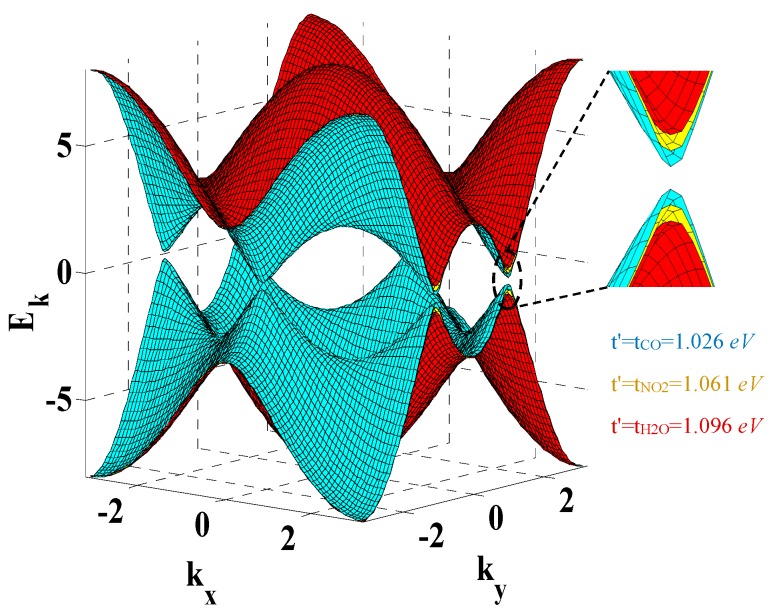
Graphene energy bandgap variation after target molecule adsorption.

To investigate the sensing properties of the graphene, we assumed the adsorption of NO_2_, CO, and H_2_O gases. Each gas molecule prefers different configurations on the graphene plane. In our study, the orientation and molecular distance from the graphene surface are considered according to work presented in [45]. On the other hand, each gas molecule has specific hopping energy when adsorbed on the graphene surface that is dependent on the type and configuration of the molecule on the substrate. After calculating the corresponding hopping energy parameter for each gas, we can apply the molecular adsorption effects on the electrical and physical properties of the graphene. The hopping energy parameter of the adsorbed molecules on a substrate can be achieved as following [50]:

(8) $$t_{\alpha \beta }=t_{R}{\left(\frac{d_{R}}{d_{\alpha \beta }}\right)}^{2}$$

where *t_αβ_* is the hopping energy parameters between the substrate and adsorbate, *t_R_* is hopping parameter between the atoms of the adsorbate (carbon atoms for graphene), *d_R_* = 1.42 Å is the carbon-carbon bond length in graphene, and *d_αβ_* represents the distance between the adsorbate and substrate. Therefore, the hopping energies for the adsorbed gases are presented in Table 1:

The adsorption effects of these three gases on the energy bandgap of graphene are investigated. The band structure analysis indicates that molecular adsorption can modify the band structure of the graphene, as illustrated in Figure 4. It can be seen that after the adsorption of CO, NO_2_, and H_2_O molecules, the energy gap of graphene shows remarkable increments, while CO and H_2_O have the lowest and highest impact on the bandgap respectively.

### 2.2. Carrier Velocity Model

When gas molecules adsorbed to the graphene surface, it modulates the energy bandgap and also the carrier concentration. The bandgap variation leads to the variation of the density of states. On the other hand, according to the literature, the velocity of carriers is a function of the density of states and carrier concentration. Now, we are going to model gas adsorption effects on the density of states and carrier concertation and study effect on the carrier velocity in the form of *I-V* characteristics. It has been reported by previous studies that the velocity of the carriers is proportional to the density of states and carrier density at any moment, as given by [25]:

(9) $$v_{av}=\frac{\int \left|v\right|D\left(E\right)f\left(E\right)dE}{n}$$

where *f(E) = 1/(1 + exp((E − E_f_)/k_B_T))* is the Fermi distribution function, *D(E)* is the density of states and *n* is the carrier concentration. The magnitude of the velocity is given as:

(10) $$\left|v\right|={\left(\frac{2(E-E_{g})}{m^{*}}\right)}^{\frac{1}{2}}$$

where m^*^ is the electron effective mass. To calculate velocity, first, we should find *D(E)* and carrier concentration. The DOS in a material describes the number of states at each energy level that can be taken by the electrons per energy interval, that is calculated as:

(11) $$D(E)=\frac{\Delta n}{\Delta E\times A}=\mp \frac{\sqrt{{\left(E_{0}+E_{0}^{\prime }\right)}^{2}-4E_{0}E_{0}^{\prime }+4t^{\prime^{2}}+36t^{2}-8k_{x}^{2}a^{2}t^{2}}}{-8\pi t^{2}a^{2}k_{x}}$$

where *A* is the graphene surface area and the wave propagation vector, *k_x_*, in the x-direction is formulated as:

(12) $$k_{x}=\frac{\sqrt{4t^{\prime^{2}}+28t^{2}-4E^{2}+4EE_{0}+4E_{0}E_{0}^{\prime }-4E_{0}E_{0}^{\prime }}}{8a^{2}t^{2}}$$

**Figure 5:**
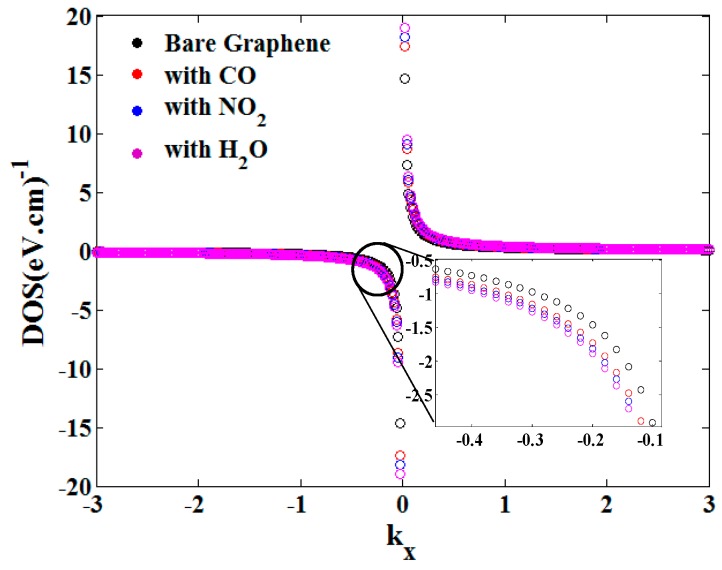
Gas adsorption effect on the graphene density of states.

The gas adsorption effect on the graphene density of states against the wave vector is illustrated in Figure 5. It can be seen that DOS changes after molecular adsorptions. On the other hand, the variation of the DOS is not the same for all gases because of the different values of the hopping energy for each gas. In addition, the DOS increases after gas adsorption caused by the bandgap opening in the graphene energy band structure induce by the molecular adsorption. Therefore, gas adsorption can change the possibility of occupying energy states by the electrons that will affect the electrical properties of the sensor. Based on Figure 5, the DOS experiences small changes against gas molecules, but a small change in DOS can lead to remarkable effects in the electrical properties of the sensor. 

The next step is to calculate the carrier concentration. The concentration of carriers is a function of the density of states and Fermi function given as [42]:

(13) n=∫D(E)f(E)dE

Based on Equations (13) and (11), we can write:

(14) $$n=\mp \int \frac{{\left({\left(E_{0}+E_{0}^{\prime }\right)}^{2}-4E_{0}E_{0}^{\prime }+4t^{\prime^{2}}+36t^{2}-8k_{x}^{2}a^{2}t^{2}\right)}^{\frac{1}{2}}}{8\pi a^{2}t^{2}k_{x}}\times \left(\frac{1}{1+e^{\frac{E-E_{F}}{k_{B}T}}}\right)dE$$

With the normalized fermi function $\eta =\frac{E_{f}-E_{g}}{k_{B}T}$ and $x=\frac{E-E_{g}}{k_{B}T}$, the final equation for the carrier concentration for the graphene, considering the gas molecule adsorption effect, could be achieved as:

(15) $$n=\mp \frac{k_{B}T}{8\pi a^{2}t^{2}}\int \frac{\sqrt{\left({\left(E_{0}+E_{0}^{\prime }\right)}^{2}-4E_{0}E_{0}^{\prime }+4t^{\prime^{2}}+36t^{2}-8k_{x}^{2}a^{2}t^{2}\right)}}{k_{x}\left(1+exp\left(x-\eta \right)\right)}dx$$

**Figure 6:**
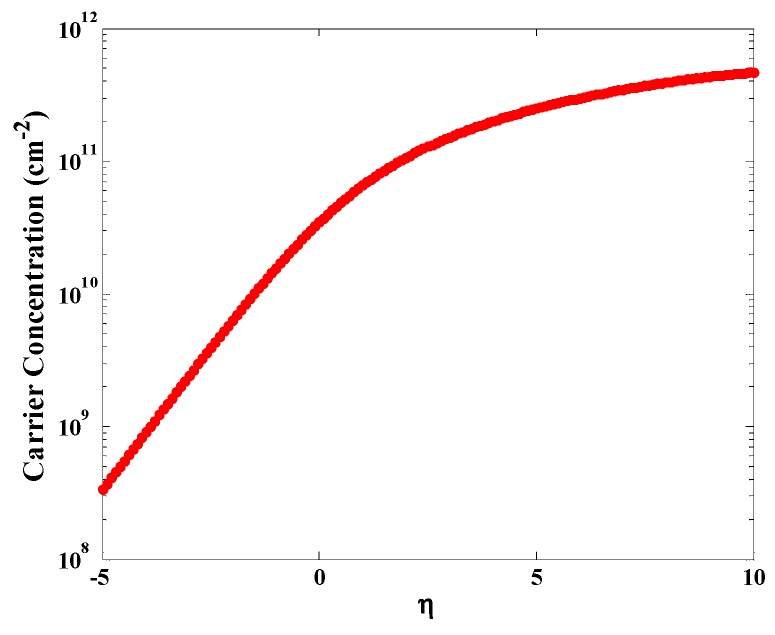
Schematics illustration of the graphene carrier concentration.

Based on Equation (15), the carrier concentration of graphene is presented in Figure 6. The concentration of the carrier is calculated to be around 10^^14^ for our graphene, which is consistent with the literature [51,52].

Now, according to Equation (11), to model the carrier velocity, the numerator is calculated as:

(16) $$\int \left|v\right|D\left(E\right)f\left(E\right)dE=\int {\left(\frac{2(E-E_{g})}{m}\right)}^{\frac{1}{2}}\left(\mp \frac{{\left({\left(E_{0}+E_{0}^{\prime }\right)}^{2}-4E_{0}E_{0}^{\prime }+4t^{\prime^{2}}+36t^{2}-8k_{x}^{2}a^{2}t^{2}\right)}^{\frac{1}{2}}}{8\pi t^{2}a^{2}k_{x}}\right)\left(\frac{1}{1+e^{\frac{E-E_{F}}{k_{B}T}}}\right)dE$$

Having *E = x.k_B_T + Eg*, normalized fermi function *ƞ*, and *x*, Equation (16) could be modified as:

(17) $$\left(\frac{\sqrt{2}k_{B}T}{8\pi t^{2}a^{2}\sqrt{m}}\right)\int \left(\mp \frac{{\left({\left(E_{0}+E_{0}^{\prime }\right)}^{2}-4E_{0}E_{0}^{\prime }+4t^{\prime^{2}}+36t^{2}-8k_{x}^{2}a^{2}t^{2}\right)}^{\frac{1}{2}}}{k_{x}}\right)\left(\frac{\sqrt{E-E_{g}}}{1+e^{x-\eta }}\right)dx$$

where *k_B_* is the Boltzmann constant and *T* is temperature. Finally, the carrier velocity for the graphene considering the molecular adsorption effects is achieved as:

(18) $$v_{av}=\sqrt{\frac{2k_{B}T}{m}}\frac{\int \frac{\sqrt{{\left(E_{0}+E_{0}^{\prime }\right)}^{2}-4E_{0}E_{0}^{\prime }+4t^{\prime^{2}}+36t^{2}-8k_{x}^{2}a^{2}t^{2}}}{k_{x}\left(1+e^{x-\eta }\right)}\sqrt{x}dx}{\int \frac{\sqrt{{\left(E_{0}+E_{0}^{\prime }\right)}^{2}-4E_{0}E_{0}^{\prime }+4t^{\prime^{2}}+36t^{2}-8k_{x}^{2}a^{2}t^{2}}}{k_{x}\left(1+e^{x-\eta }\right)}dx}$$

**Figure 7:**
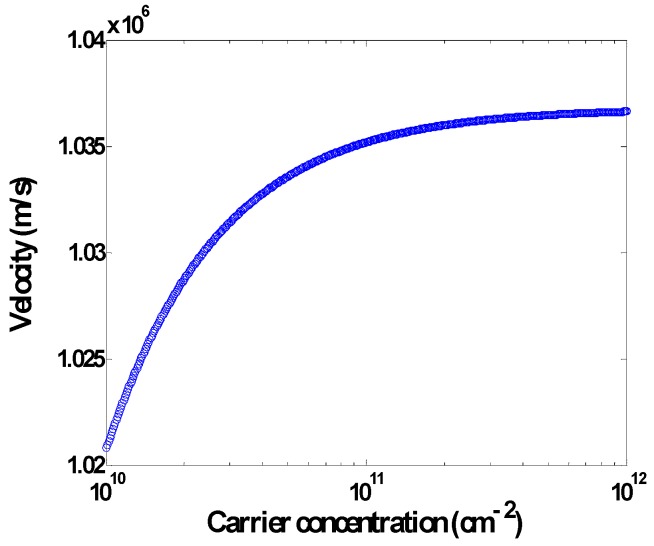
Carrier velocity of the graphene against the carrier concentration.

The parameters *t’* and $E_{0}^{\prime }$ exert the gas adsorption effects on the velocity of carriers. Based on Equation (18), the velocity of the carriers for the bare graphene is depicted in Figure 7.

The investigation of the carrier velocity variation after the gas adsorption is performed in the form of current-voltage analysis. Hence, we need to obtain the *I-V* characteristics based on the carrier velocity. Based on the relation between *I-V* and velocity, the *I-V* can be written as a function of the carrier velocity:

(19) $$I_{ds}=nqA\left(\sqrt{\frac{2k_{B}T}{m}}\frac{\int \frac{\sqrt{{\left(E_{0}+E_{0}^{\prime }\right)}^{2}-4E_{0}E_{0}^{\prime }+4t^{\prime^{2}}+36t^{2}-8k_{x}^{2}a^{2}t^{2}}}{k_{x}\left(1+e^{x-\eta }\right)}\sqrt{x}dx}{n}\right)$$

(20) $$I_{ds}=qA\sqrt{\frac{2k_{B}T}{m}}\int \frac{\sqrt{{\left(E_{0}+E_{0}^{\prime }\right)}^{2}-4E_{0}E_{0}^{\prime }+4t^{\prime^{2}}+36t^{2}-8k_{x}^{2}a^{2}t^{2}}}{k_{x}\left(1+e^{x-\eta }\right)}\sqrt{x}dx$$

where *A* represents the surface area, which carriers travel through it, *n* represents the carrier concentration and *q* is the electrical charge. Finally, based on Equation (20) the electrical properties of the gas sensor can be investigated. 

**Figure 8:**
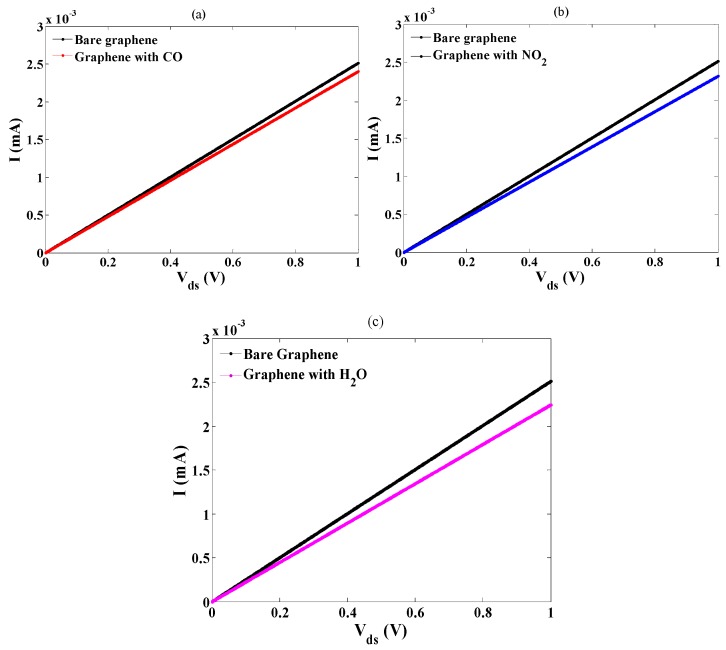
The *I-V* analysis of the gas sensor after (**a**) CO adsorption; (**b**) NO_2_ adsorption; (**c**) H_2_O adsorption.

The *I-V* characteristics of the gas sensor in the exposure of CO, NO_2,_ and H_2_O are plotted, as shown in Figure 8. According to Figure 8, the current has decreased after molecular adsorption, and the sensor exhibits different properties against each target gas molecule. Upon gas molecules adsorption on the graphene, two phenomena occur that alter the current of the sensor. The atomic forces between graphene and adsorbed molecules induced by the covalent bonds, exert some changes to the graphene structure, and results to the variation of the band structure and energy bandgap of graphene and therefore, modifies its electrical conductivity and density of states and subsequently current-voltage properties.

**Figure 9:**
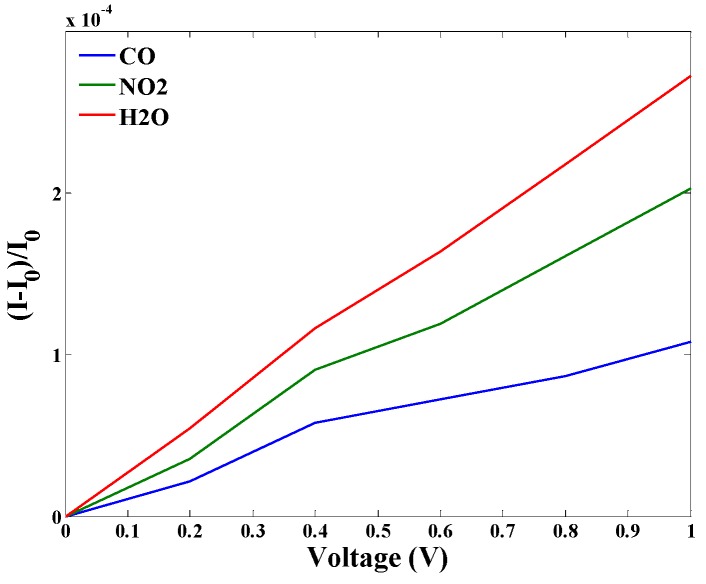
The response of the GFET based sensor toward CO, NO_2_, and H_2_O.

In addition, the transfer of charge between substrate and adsorbate changes theG carrier concentration on the graphene surface. The variation of DOS and carrier concentration affect the velocity of the carriers that lead to change the *I-V* of the sensor. After gas adsorption, the graphene energy bandgap increases, thus, the conductivity decreases and reduces the current. Moreover, the response of the gas sensor against adsorbed molecules is calculated and presented in Figure 9. It can be seen that the response of the sensor is the highest for the H_2_O and the lowest for the CO molecules. This result is consistent with the band structure and *I-V* analysis as we saw more bandgap increment and current decrement after H_2_O adsorption than other molecules.

For future investigations and further evaluation of the models, the comparison study with the results of similar studies such as first principle calculations or experimental data can be conducted, as we couldn’t find similar work at the present time. However, according to the archived results and trend of the plots, the proposed models illustrate reasonable performance. In addition, the range of values of the IV, velocity, band structure, and other plots indicates reasonable quantities and, thus the proposed models seem to be valid. 

## 3. Conclusions

In this study, a series of analytical models for the detection of the gas molecule using a platform based on graphene FET is developed and applied for CO, NO_2,_ and H_2_O detection. Based on the results, it was shown that the energy dispersion relation of the graphene varies when exposed to the gas molecules. The DOS and carrier concentration parameters were calculated, and the variation of the DOS in after molecular adsorption of the gases were monitored. Based on the DOS and carrier concentration models, the carrier velocity model was developed and analyzed in the form of current-voltage properties. The *I-V* analysis indicates that the current of the sensor has changed and decreased after the adsorption of the gas molecules. After gas adsorption, the graphene energy bandgap changes that affect the conductivity of the graphene and increase the channel resistance. On the other hand, after molecular adsorption, the change transfer between molecules modifies the number of the carriers and hence the velocity of the charged carriers on the graphene plane that leads to modulate the *I-V* characteristics. It can be seen that the most and the least current reduction have occurred after H_2_O and CO adsorption, respectively. This implies that the response of the sensor is not the same for different gases. Finally, the main outcomes of this study can be mentioned as: (i) the analytical formulation, which allows the convenient use of the results for simulators, (ii) the demonstrated selectivity of the sensors with respect to different gases.

## Figures and Tables

**Table 1:** The calculated hopping energies for the adsorbed gas molecules.

Adsorption Type	Distance from Graphene Surface	Hopping Parameter
H_2_O	*d_αβ_* = 3.5 Å	*t_C-H2O_* = 0.406 t_R_
NO_2_	*d_αβ_* = 3.61 Å	*t_C-NO2_* = 0.393 t_R_
CO	*d_αβ_* = 3.74 Å	*t_C-CO_* = 0.38 t_R_
